# On the Deaths of Some Eminent Persons of Modern Times

**Published:** 1835-07-01

**Authors:** 


*On the Deaths of some eminent Persons of Modern Times.* By Sir Henry Halford, Bart., M.D., G.C.H., President of the Royal College of Physicians.—London, 1835. Small 8vo. pp. 40.

The present essay is conspicuous for the same elegance of style, and the same medical tact, which have distinguished all the writings of the learned President of the College of Physicians: the subject, too, is a particularly happy one. Medicine, essentially a tentative art, owes everything to its history, without which it is a rough and destructive empiricism; for, untaught by registered failures, the despiser of learning copies, without knowing it, the mistakes of his predecessors. Medicine, again, throws great light on general history. The loss of a battle, the infraction of a treaty, the hasty resolves of a popular chief, must often have arisen from a dyspepsia or a neuralgia: as Pope says, "Perhaps the man was sick, or had not dined."

Sir H. Halford begins his catalogue of the illustrious dead with Henry VIII. and Cardinal Wolsey.

"That prince, when he ascended the throne at the age of twenty, is said to have been one of the handsomest and most portly men of his time. In proof of his comely looks in his early manhood, I may refer you to Holbein's pictures of him at Windsor, and more especially to that whole-length portrait of him at Belvoir Castle; and, as to his stature, I may argue from the remains of him which I have seen in his coffin, and from his favourite large arm-chair, which is still preserved in the corridor at Windsor Castle. As life advanced, however, he became corpulent, unwieldy, and gross in his habit, was covered with sores, and died of a dropsy at the age of fifty-six.

"Henry's state of health, in the decline of his life, made him a great dabbler in physic; and the King not only offered medical advice on all occasions which presented themselves, but made up the medicines himself, and administered them. We find in that curious magazine of materials for history, the British Museum, a volume containing a large collection of recipes for plasters, spasma draps (dipped plasters), ointments, waters, lotions, and decoctions, devised and made by the King himself and his physicians, applicable, perhaps, amongst other diseases, to that which had been imported, some twenty-five years before, from Naples; and, in Sir Henry Ellis's most interesting collection of Original Letters, we read one from Sir Bryan Tuke to Cardinal Wolsey, giving an account of an interview with the King, in which his Majesty prescribed for Sir Bryan, and sent also some excellent instructions to Cardinal Wolsey, how he might avoid the infection of the sweating sickness, and how he should treat the disease should it attack him.*

"If the Cardinal had devoted some of the leisure which he could spare from the duties of his great offices to medical pursuits, we should not have been surprised, recollecting that almost all the learning of the land was confined to the ecclesiastics at that time, and that it was the province of the bishops, in their several dioceses, to license medical men to practise physic, before that duty was assigned exclusively to the College of Physicians.

"The Cardinal died of a broken heart, in fact, though the

* "This singular malady prevailed five times epidemically in England, producing a great mortality here, and was imported originally, it should seem, by King Henry VIIth's army, when it landed at Milford Haven. The best account of it is given by Dr. Kay, successor of Linacre, as president of this College, and founder of Caius College in Cambridge."

symptoms which afflicted him in the last days of his life were those of a dysentery.

"It is impossible to read his secretary's (Mr. Cavendish) account of his life, without being deeply interested in the fate of that great man, and without feeling assured that the calumnies heaped upon him so unsparingly, after he had lost the King's favour, and had been despoiled of all his property, without a judicial inquiry into his malversations, whatever they might be, were most grossly exaggerated. But we know how easy a topic it is to dwell upon the faults of departed greatness.

> 'Sejanus duciter unco
> Spectandus; gaudent omnes; quæ labra, quis illi
> Vultus erat? nunquam, si quid mihi credis, amavi
> Hunc hominem.'
> 'Sed quid
> Turba Remi?'

Human nature is always the same:

> 'Sequitur fortunam, ut semper, et odit
> Damnatos;'— (*Juvenal*, Sat. 10.)

and those who had watched 'the sign to hate,' in the words of the great moralist, now blackened the Cardinal with the most malignant reproaches and imputations. Hollingshed has it, and Shakspeare copies from Hollingshed, that 'of his own body he was ill, and gave the clergy ill example;' and the 6th and 38th charges in the articles of impeachment preferred against him may have furnished this remark to the chronicler. But be it remembered that these charges were not only never proved, but were absolutely rejected by the House of Commons; and that he was on his way to London, to refute all these disgraceful calumnies, when he was seized with dysentery, and died at Leicester.

"It appears that the Earl of Shrewsbury, at whose house he had been entertained on his road from Yorkshire, seeing him low and dejected, and most anxious for an opportunity of justifying himself, had prevailed upon the King to send down Sir William Kingston, governor of the Tower, with a proper guard of honour, to conduct him to London. Sir William, in his humanity, encouraged the Cardinal to think better of his health than he was disposed to do, and spake cheeringly to him. The Cardinal replied, 'Nay, in good sooth, Master Kingston, my disease is such that I cannot live; for I have had some experience in physic. Thus it is: I have a flux with a continued fever, the nature whereof is, that if there be no alteration of the same within eight days, either must ensue excoriation of the entrails, or delirium, or else present death. And, as I suppose this is the eighth day, and if ye see no alteration in me, there is no remedy. Death, which is the least of these three, must follow. Farewell! I can say no more; but wish, ere I die, all the King's affairs to have good success. My time draweth on fast: I may not tarry with you. But forget not what I have said and charged you withal; for, when I am dead, ye shall peradventure remember my words better.' Incontinent, the clock struck eight,

'The very hour himself foretold should be his last.'

*Shakspeare.*

The hour at which he knew, and had prophesied, he should die, he gave up the ghost, and thus departed this present life.

"Mr. Cavendish remarks, that 'whatsoever any man hath conceived in him, whilst he lived, or since his death, thus much I dare be bold to say, without displeasure to any person, or of affection, that, in my judgment, I never saw this realm in better obedience and quiet than it was in the time of his rule and authority, nor justice better administered with indifferency.'

"And, as I have quoted Shakspeare to his disparagement, let me add from the same, and after the poet's example, what he has said to his credit.

'His overthrow heap'd happiness upon him,
For then, and not till then, he felt himself,
And found the blessedness of being little;
And, to add greater honours to his age
Than man could give him, he died fearing God.'"

Edward VI. died of pneumonia after measles.

"Mary, the elder daughter of King Henry VIII., inherited from Queen Catherine, her mother, a weak constitution, and was always of feeble and unpromising health. When she arrived at mature age, the peculiarities of her sex were irregular and deficient; for which were prescribed frequent bleedings and exercise on horseback. After her marriage with Philip of Spain, she referred this irregularity to pregnancy; and died at last of dropsy. It appears in Sir Frederick Madden's introductory memoir to the privy purse of Queen Mary, that she was bled very frequently, and that fees were paid again, and again, and again to the surgeon who bled her; till at last she grew so pale, as to convey, even to unprofessional eyes, a conviction that she laboured under an internal organic disease; in which, probably, the better practice of modern days, by chalybeates and aloetic medicines, would not have availed her Majesty more than the repeated bleedings and horse exercise had done." (P. 13.)

Dr. Bates, in his "Elenchus Motuum Nuperorum in Angliâ," has given an account of the last sickness of Oliver Cromwell. Post-mortem examination showed that the chief disease was in the spleen, which was filled with matter like the lees of oil.

Charles II. died of apoplexy.

"It appears that the king had just risen from his bed, at eight in the morning, when he felt an unusual sensation in his head. Shortly after complaining of this he fell down speechless, and without the power of motion. A medical gentleman of the army happened to be waiting in the next room to assist his Majesty in making some experiments for the fixing of mercury, who most properly thought himself justified in taking away sixteen ounces of blood, even before he had summoned the King's physicians. When they arrived, they commended his decision, and followed up the first step by cupping his Majesty to eight or ten ounces more. They ordered, moreover, an antimonial emetic (but little of which could be got down), a powerful purgative, and clysters. These expedients producing little or no effect, a blister was applied to the King's head, and other remedies were prescribed to which we have recourse in modern practice. But all in vain. The King lingered four days, during which it is palpable, from the prescriptions, that not only no improvement took place, but everything proceeded from bad to worse, from hour to hour, until his Majesty expired, in the fifty-fourth year of his age.

"His brother and successor, the Duke of York, afterwards King James II., 'summa in regem pietate, et plusquam fraterno amore affectus,' as the narrative states, watched the symptoms most anxiously, and hardly ever left the sick room, 'ut omnibus constiterit maluisse ipsum charissimi Fratris consortio perfrui quam sceptro.'

"Had there been safety in a multitude of counsellors, the King's life must have been preserved, for I perceive the signatures of not less than fourteen physicians to one of the prescriptions, amongst whom were Sir Charles Scarborough, Doctors Lower, Charlton, Millington, Wytherly, and others, with whose portraits you are familiar in the room below, and who have left you an ample inheritance of fame.

"Their materia medica seems to resemble much that in use at this time of day, save that we have improved upon the spiritus cranii humani, twenty-five drops of which were ordered in a cordial julep, 'ad refocillandas Regis vires,' (when his Majesty was sinking,) by substituting for this bad and disgusting sal volatile, a more effectual preparation of the stag's horn. This calls to my recollection an original prescription, which was shown me, in which a portion of the cranium humanum was ordered in a powder for Sir Nicholas Throckmorton, in the time of the protectorate. It was found on a file of prescriptions dug out of the ruins of a house in Duke-street, Westminster, said to have been inhabited by Oliver Cromwell's apothecary." (P. 18.)

There was effusion of lymph in the ventricles, and at the base of the brain; and an old adhesion of the lungs to the right pleura.

William III. died from the effects of a fall from his horse, which broke through an old adhesion of the lungs to the pleura, occasioning inflammation, suppuration, and death.

His consort, Mary, died of the smallpox: Burnet censures Dr. Radcliffe's treatment of her case, but, in our author's opinion, without reason.

Dryden died of gangrene of the foot, probably depending on ossification of the arteries.

As it would hardly be fair to take more from a short essay, we must unwillingly omit the other cases, (Swift; George I. II. and III.; and the Duke of Gloucester;) and give only the just and tolerant conclusion.

"I will now thank you, gentlemen, most respectfully for your attention, and only entreat you to read history,—not with that total disbelief of it which Sir Robert Walpole is said to have expressed, when a volume of history was offered him for his amusement, after his retirement from public life,—but with some mistrust and reserve, recollecting how difficult it is to develope the motives of human conduct; how easily the spirit of party insinuates itself into the historian's mind, and colours his narrative; and how almost impossible it is for an unprofessional writer to appreciate fully the effect of diseases of the body upon the minds and actions of men." (P. 40.)

---

*A Dictionary of Practical Medicine.* By JAMES COPLAND, M.D. F.R.S. &c. Part III.—London, 1835. 8vo. pp. 320.

